# Bipolar resection of the bladder and prostate – Initial experience with a newly developed regular sized loop resectoscope

**Published:** 2009-11-25

**Authors:** T Bach, TRW Herrmann, C Cellarius, B Geavlete, AJ Gross, M Jecu

**Affiliations:** * Asklepios Hospital Barmbek, Department of Urology, HamburgGermany; ** Hannover Medical School, Department of Urology, Hannover Germany; *** ‘Sf. Ioan’ Clinical Emergency Hospital, Department of Urology, BucharestRomania

**Keywords:** Bipolar transurethral resection, regular sized loop, cutting speed, tissue carbonization, TURP, TURBT

## Abstract

**Objective**: Bipolar transurethral resection (TUR) has been introduced in the clinical practice nowadays. Benefits from bipolar TUR are represented by the use of saline irrigation, which avoids hypoosmotic hyperhydration (TUR–Syndrome), as well as by the reduced risk of obturator nerve stimulation. However, the previously introduced smaller bipolar resection loop caused prolonged operating–time. We report our initial experience with a newly developed regular sized loop for a bipolar resectoscope.

**Materials and Methods**: Different loop calibers and configurations were tested and compared to a previously introduced bipolar system and conventional resection devices in TUR of benign prostate hyperplasia (BPH) and bladder tumors (TURP and TURBT). The resected tissue was pathologically examined for thermal damage and compared to a control group of monopolar conventionally resected tissue.

**Results**: The handling of the resectoscope was comparable to that of the conventional ones. Cutting control, cutting speed and coagulation effectiveness were excellent, and no obturator nerve stimulation occurred. The resection area could easily be assessed and tissue examination showed no differences in terms of quality and quantity of thermal damages, since tissue carbonization was reduced. There was no sticking of the resected tissue on the loop.

**Conclusion**: Regular sized loop bipolar resection is safe and efficient. Coagulation and cutting extent control seem superior to conventional TUR. Due to reduced carbonization, the resection ground can be easily assessed. The risk of obturator nerve stimulation is reduced. The histological quality of the tissue is not impaired. This device combines the advantage of a regular size resection loop with bipolar resection in normal saline. It has the potential to become a valuable alternative to monopolar TUR.

## Introduction

Dating TUR with a high frequency current in a non-conductive medium has been the standard treatment for transurethral therapy of bladder tumors and BPH. The current passes from the resection loop through the patient to a neutral electrode. This can lead to potential complications. The excessive uptake of the anionic non–conductive irrigation fluid can lead to hypoosmotic hyperhydration, which may cause TUR syndrome. An electrical stimulation of the obturator nerve may lead to spontaneous contraction of the adductor muscle and subsequently to bladder perforation.

In order to overcome these problems, bipolar TUR has been introduced. Due to the modified current flow, the use of a non–conductive irrigation fluid became unnecessary, and it was replaced by normal saline, thus theoretically eliminating the TUR–Syndrome [1]. 

The current flow is modified and passes from the resection loop through the conductive irrigation fluid to the metal resection sheath, an additional loop or an extra shackle. Since the impedance of the patient is 10–fold higher than that of the irrigation fluid, the patient no longer constitutes a direct part of the current circle [2]. Therefore, the risk of obturator nerve stimulation is significantly reduced. Problems with the previously introduced bipolar systems occur due to technical reasons, such as smaller and thinner resection loops causing prolonged operating time [3].

We report our initial experience with a newly developed bipolar resectoscope (S(a)–Line, Richard Wolf, Germany), provided with a regular sized resection loop.

## Material and methods

All operations were carried out by a single surgeon. The results were compared: bipolar regular sized loop TUR to previously introduced bipolar resectoscopes and to conventional TUR using a monopolar system. 

**Fig 1:**
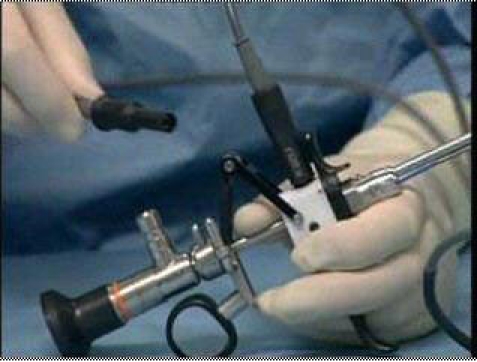
The Wolf S(a)–Line System

The new Wolf S(a)–Line System (26 French resectoscope with continuous–flow irrigation sheath– Fig 1) was compared to a conventional monopolar system consisting of a 27 French resectoscope (Olympus OES 4000), using the commercially available sorbit–mannit–solution as irrigation fluid.

**Fig 2:**
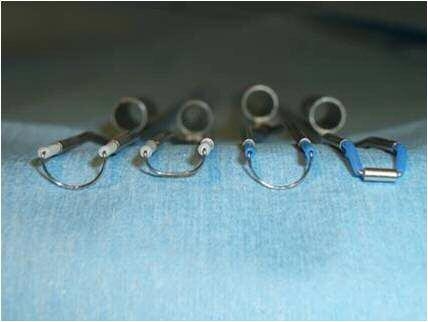
Different types of loop calibers and configurations

**Fig 3:**
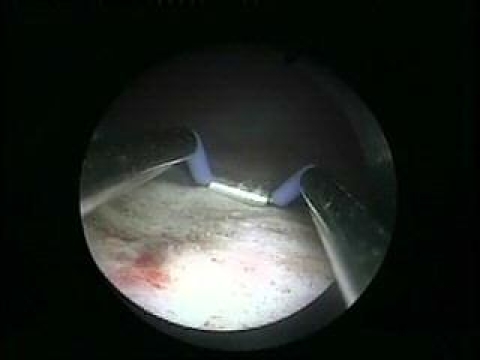
The 27 French TURis system (Olympus OES Pro)

Furthermore, it was also compared to the previously introduced 27 French TURIS system (Olympus OES Pro), presenting a smaller resection electrode (Fig 3). All operations were carried out under intravenous anesthesia, without muscle relaxation or blockage of the obturator nerve. Different types of loop calibers and configurations were tested and used in the resection of bladder tumors and BPH (Fig 2). All operations were carried out by using an ‘Erbe VIO’ generator.

The resected tissue was pathologically examined and categorized in terms of quality and quantity of thermal damage produced to the resection ground. The classification of the coagulation artifacts was performed after conventional dying of all resection chips in haematoxylin–eosin–dye. The quality of the hermal artifacts was divided into three grades (Table 1).

**Table 1:** Pathological grading

Degree of thermal damage	Characterization
0	No thermal damage
1	Lowest grade of thermal artifacts. The cellular structure is identifiable and not impaired.
2	Medium grade. Cellular structure and nuclei are impaired, but still identifiable.
3	High grade artifacts. Complete loss of the cellular structure. No differentiation of the cellular parts.

A total of 18 patients were included in this preliminary trial. The patients' age and the distribution of bladder tumors or BPH were comparable. All operations were performed without any complications. The handling was comfortable and comparable for all tested resectoscopes. 

**Chart 1:**
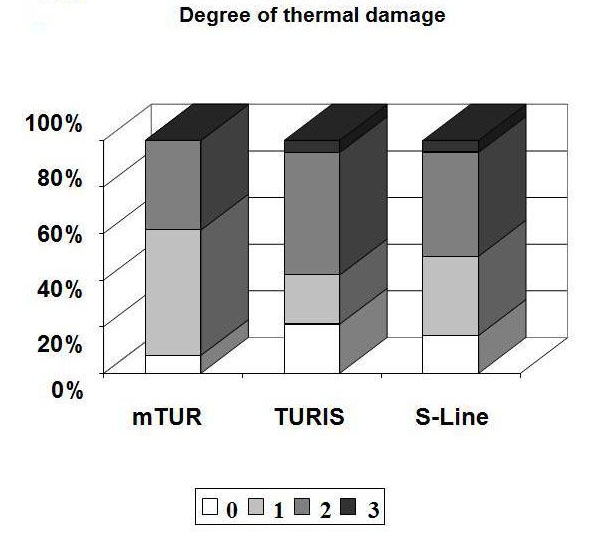
Degree of thermal damage

Regardless of the operated organ (bladder, prostate), the histological examination of the resected tissue showed no significant differences regarding the quantity as well as the quality of the thermal damage in any group (Table 2, Chart 1). In all cases, the assessment of the tumor stage and grade was possible.

**Table 2:** Degree of thermal damage

	Monopolar TUR	TURIS	**S–Line**
Grade 0 [%]	7,7	21,1	**16,3**
Grade 1 [%]	53,8	21,1	**34,0**
Grade 2 [%]	38,5	52,6	**44,6**
**Grade 3 [%]**	**0**	**5,2**	**5,1**

The operating time was comparable between the standard monopolar and the S(a)–Line resectoscopes. Regarding the previously introduced bipolar device (TURis), the resection time remains longer, (Table 3), mainly due to the smaller diameter of the loop (Fig 3).

**Fig 4:**
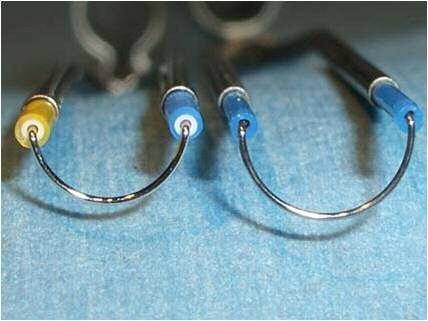
Carbonization of the resected area

The new Wolf resectoscope provided constant cutting speed and control, combined with effective coagulation. Carbonization of the resected area is reduced (Fig 4).

No sticking of the resected tissue on the loop occurred. Stimulation of the obturator nerve was not recorded. From the surgical point of view, the beginning of a cut was comparable to conventional TUR.

**Table 3:** Operation characteristics

		TURP	>
**Volume**	31	28	**27**
**Time**	43	54	**45**
**N**	3	3	**2**
		TURBT	
**Time**	18	21	**19**
**N**	**4**	**3**	**3**

## Discussion

Monopolar TUR is considered the gold standard in the surgical treatment of BPH and non–muscle invasive bladder tumors. Bipolar TUR has been introduced as a potential alternative to conventional TUR by using a monopolar electrocauterization system. The bipolar resection systems use 0.9% saline solution as irrigation fluid, which theoretically eliminates the risk of TUR syndrome [1]. 

The current does not pass through the patient, as it travels from the active electrode through the irrigation fluid to a negative return electrode. This negative electrode varies from manufacturer to manufacturer. It consists of an extra loop, an extra shackle or the metal irrigation sheath [4]. The modified current flow significantly reduces the chances for obturator nerve stimulation, and subsequently, the risk of bladder perforation due to spontaneous contraction of the adductor muscle [2, 5].

While cutting, the conductive irrigant is converted into a plasma layer around the resection loop, which provides accurate dissection and efficient coagulation, together with a significant reduction of the carbonization process. The plasma layer also avoids the sticking effect of the resected tissue on the loop [4].

The advantages offered to the surgeon during the bipolar TURP, consisting mainly of a better cutting capacity and reduced adherence of fragments, are quoted in various articles [6]. This method was successfully applied even in patients with large prostate glands and significant comorbidities [7]. 

Compared to monopolar electrocautery, bipolar resection devices seem to reduce intraoperative bleeding in an ex-vivo setting [5]. Bipolar TUR was successfully used in pregnant women, without postoperative fetal repercussions [8], as well as in patients with an implanted cardioverter defibrillator, which was not deactivated before resection [9]. Bipolar TURP manages to put an end to the disadvantages of bipolar transurethral vaporization of the prostate, which consist of postoperative irritative urinary symptoms, absence of histology, and rather temporary clinical outcomes. It provides the patients with reduced catheterization time and hospital stay [10].

The coagulation depths achieved using the mono– and bipolar TURP proved to be greater than the mean diameter of prostatic microvessels. Moreover, the mean coagulation depth specific to monopolar TURP was described as being smaller than the maximum microvessel diameter, and both of them have been over ceded by the bipolar TURP mean coagulation depth. That is to say that the haemostatic capability of bipolar TURP is significantly improved in comparison with monopolar TURP [11].

The disadvantages of the newly introduced bipolar resection devices occur mainly due to the smaller resection loop, which causes prolonged operating times, especially in cases of larger resection volumes (> 25 gr.) [3].

As far as bladder tumors are concerned, bipolar electrocautery was emphasized as a suitable instrument for TURBT, providing bladder tissue samples of the same histological value as those obtained from standard monopolar resection. However, the bladder tumor chips obtained with bipolar TURBT were smaller due to the reduced size of the bipolar loop [12]. This situation may prove significantly important especially in large bladder tumors, leading to an important increase of the resection time. 

The bipolar TUR was also described as a promising therapeutic method for the surgical treatment of bladder outlet obstruction. However, for this particular type of bipolar resection as well, the operating time was significantly longer compared with the monopolar one [13]. The already available bipolar systems showed difficulties during the beginning of the cut, especially in previously resected tissues [2, 3]. Different authors describe a slight prolongation regarding the initiation of the cut [14].

In this trial, a newly developed bipolar system with a regular sized resection loop has been tested and compared to conventional monopolar TUR and a previously introduced bipolar resection system (TURIS). The new Richard Wolf S(a)–Line combines the advantages of a bipolar resection system with the larger loop of a conventional TUR system.

The cutting speed, cutting control and coagulation effectiveness were excellent. No differences were found regarding the beginning of a cut, while comparing a fresh tissue area with a previously resected one. Moreover, there were no significant differences between the Wolf resectoscope and the conventional monopolar system.

The histological examination of the resected tissues showed no significant differences in terms of quality or quantity of thermal damage for all three devices. Determining the stage and grading of the resected specimens was possible in all cases.

## Conclusion

The S(a)–Line resectoscope combines the advantages of monopolar TUR (larger loop, shorter operating time, satisfactory cutting performance) with the advantages of a bipolar resection system (0,9% saline solution as irrigation fluid, no TUR syndrome, reduced risk of obturator nerve stimulation). 

Although the new system has yet to be tested in larger series of patients, it has the potential to become a valuable alternative in the transurethral resection of bladder and prostate.
